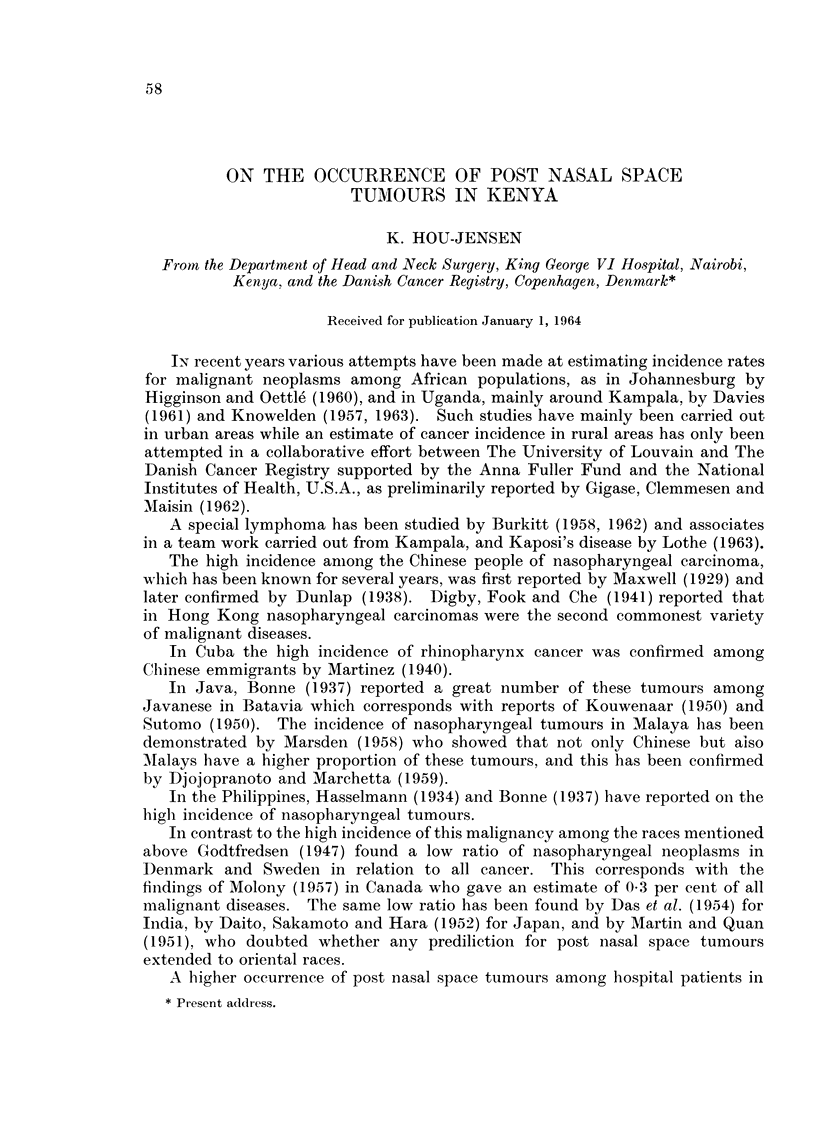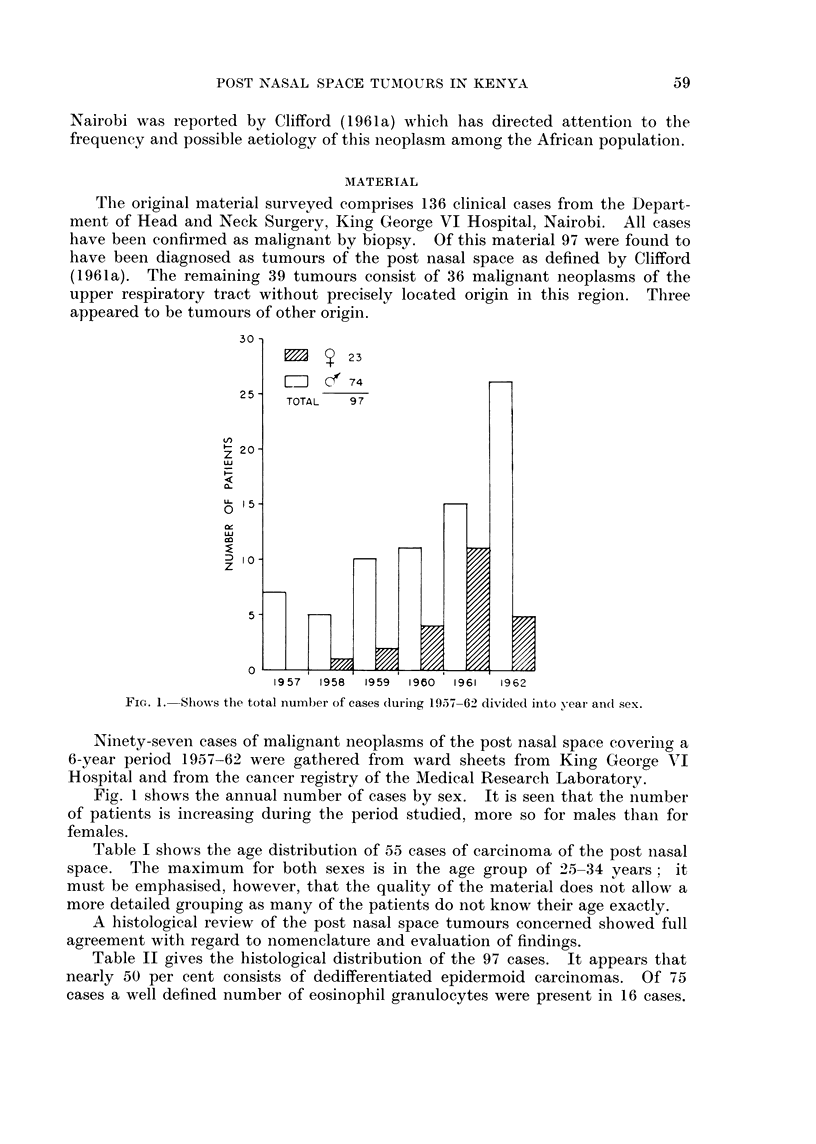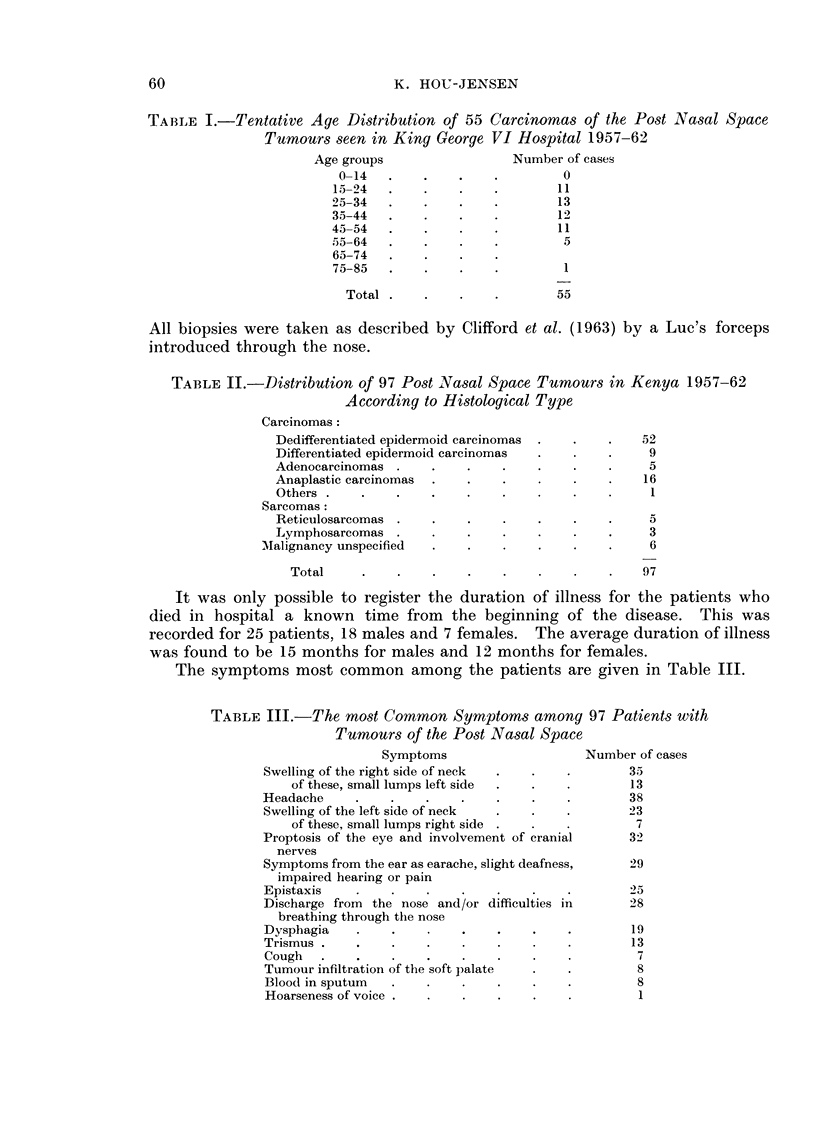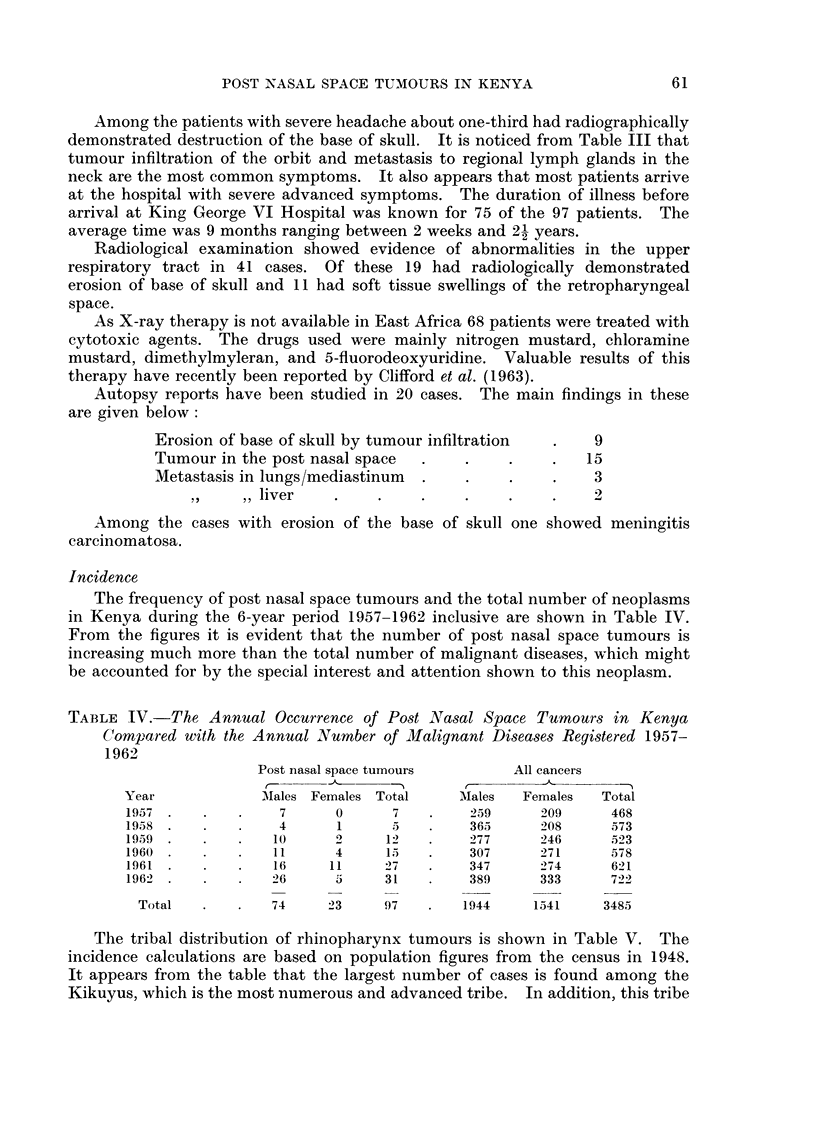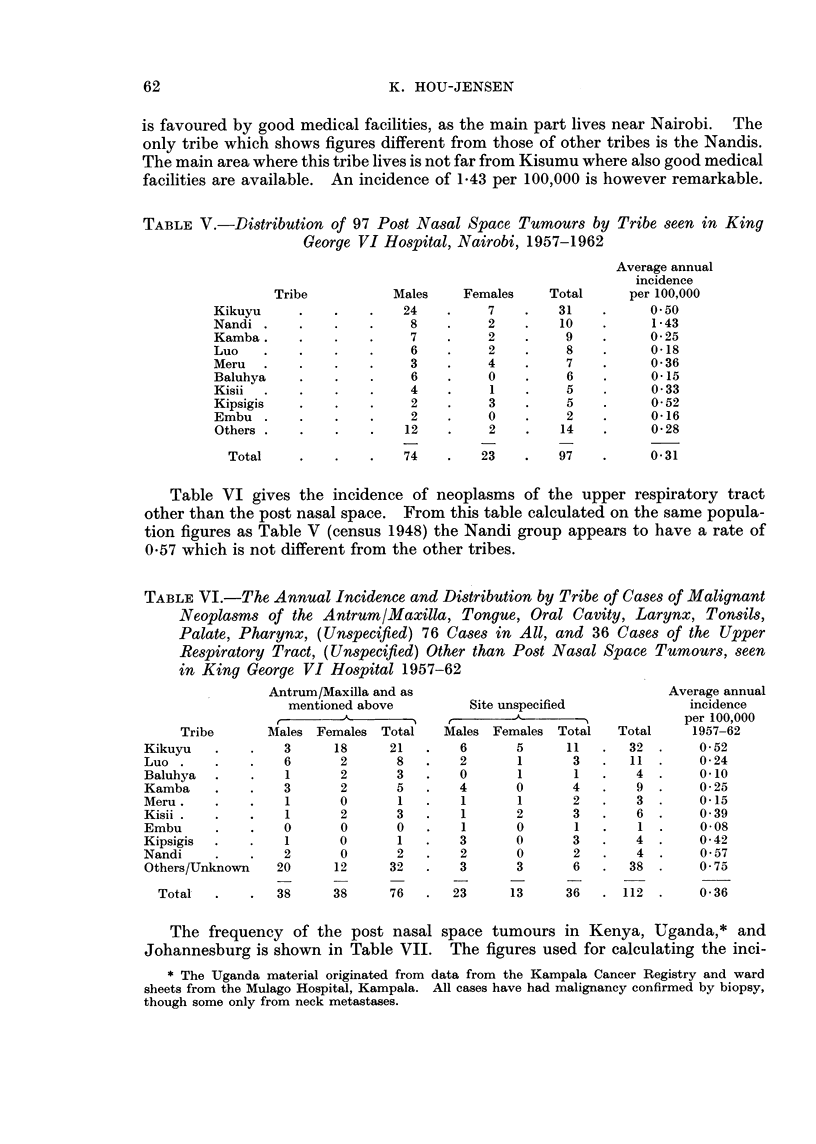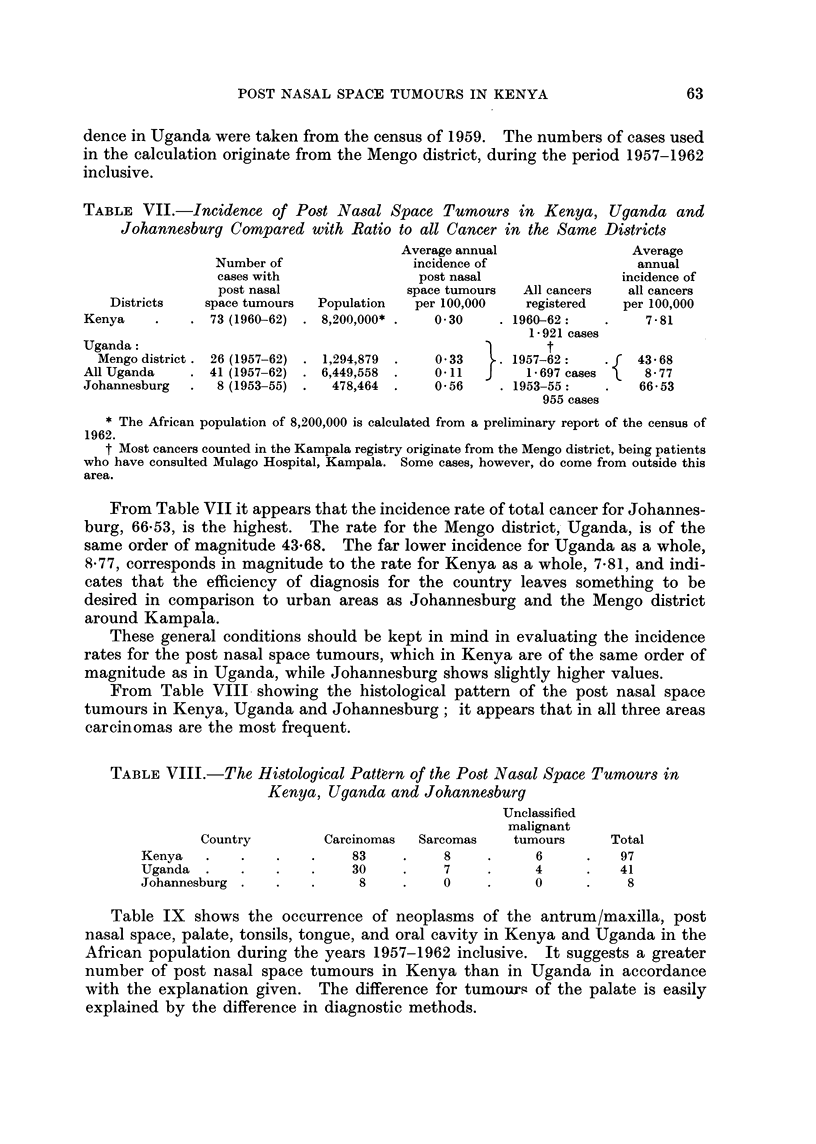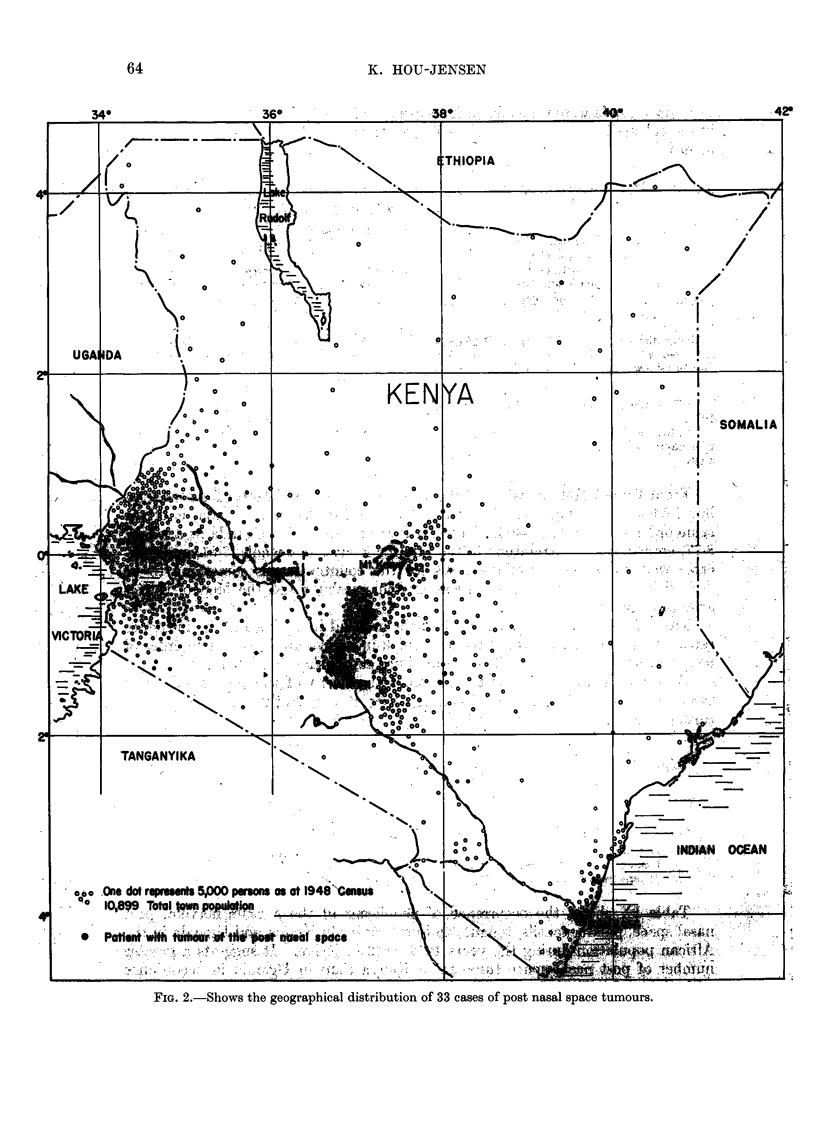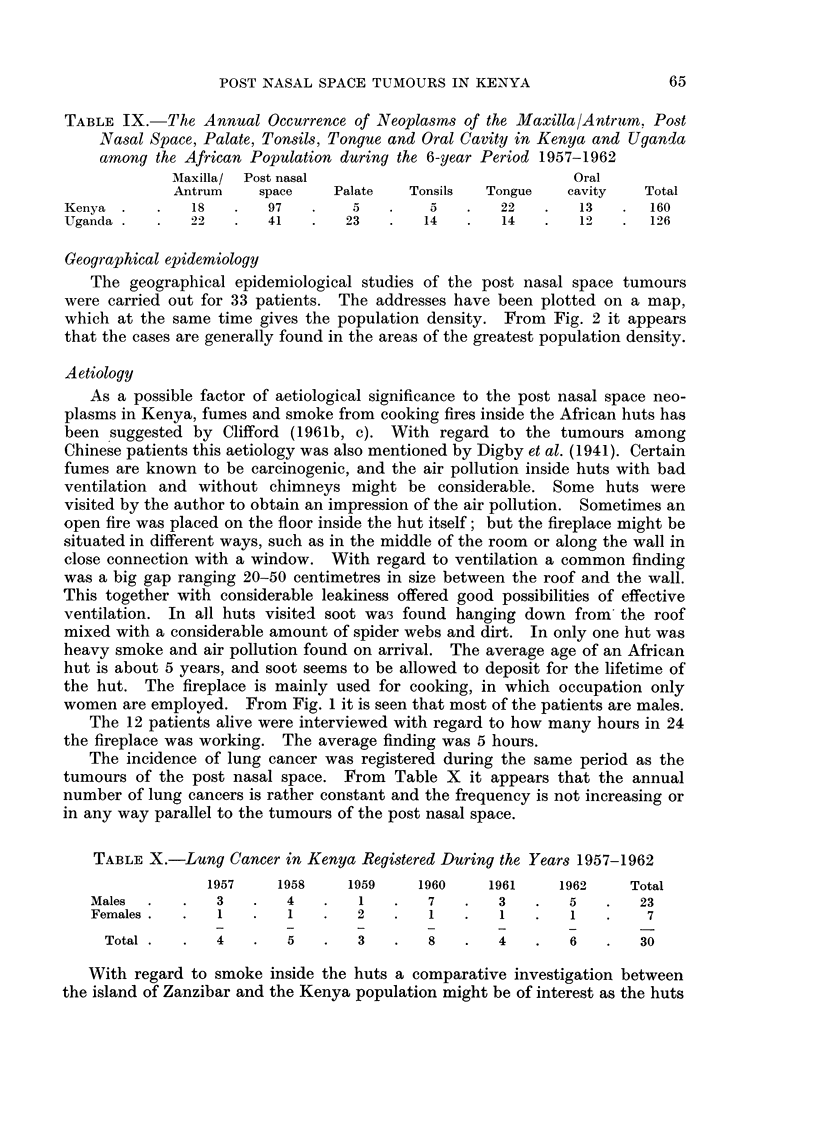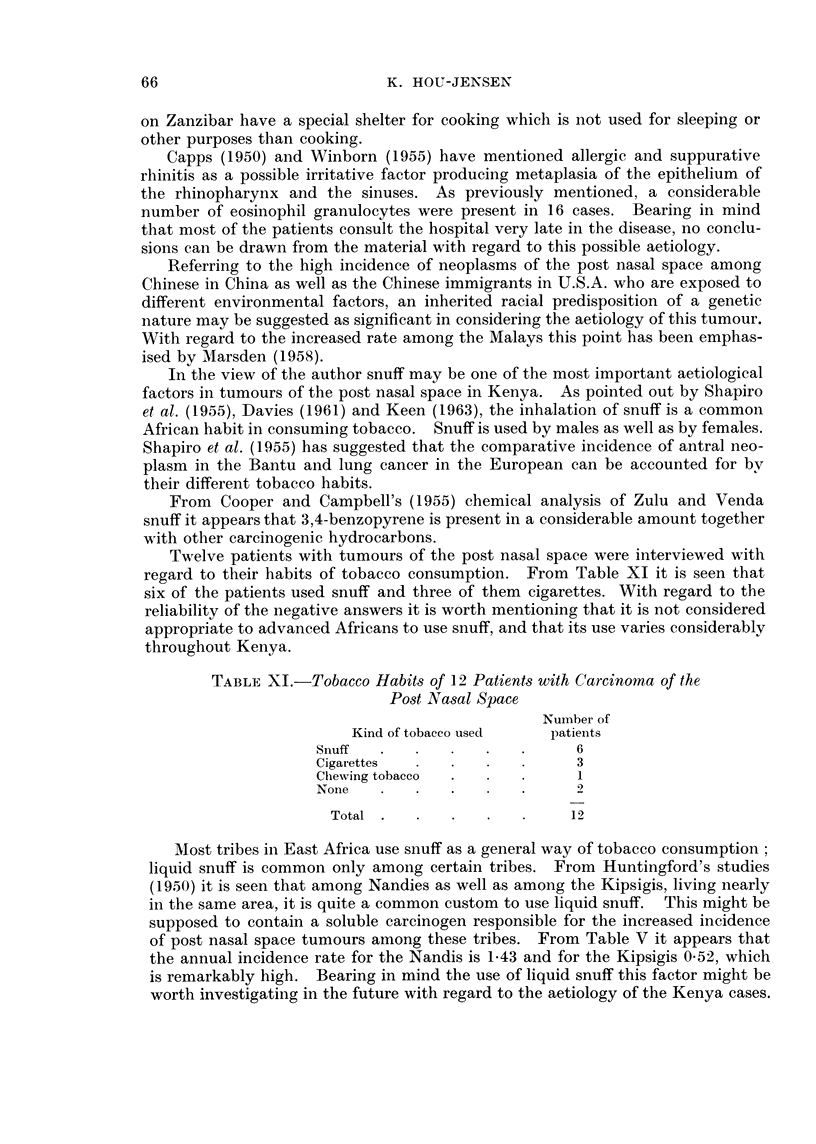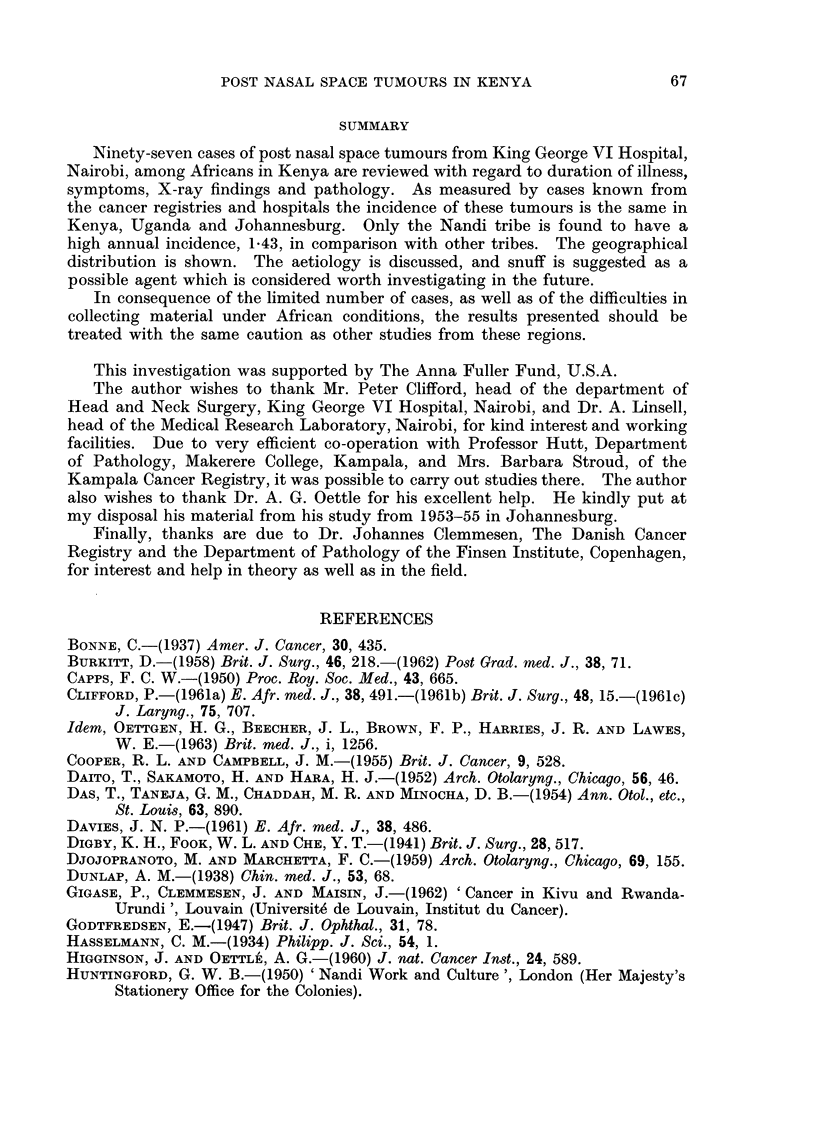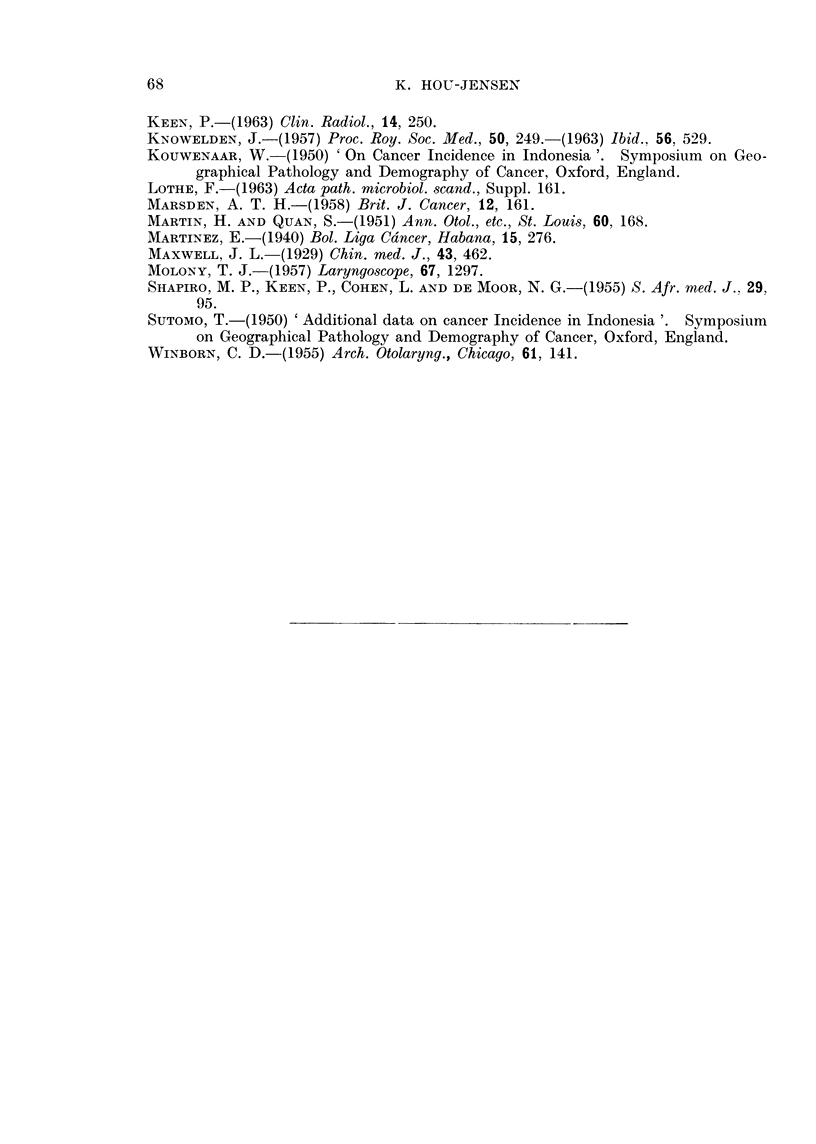# On the Occurrence of Post Nasal Space Tumours in Kenya

**DOI:** 10.1038/bjc.1964.6

**Published:** 1964-03

**Authors:** K. Hou-Jensen


### ON THE OCCURRENCE OF POST NASAL SPACE TUMOURS IN KENYA

K. HOU-JENSEN

*From the Department of Head and Neck Surgery, King George VI Hospital, Nairobi, Kenya, and the Danish Cancer Registry, Copenhagen, Denmark**

Received for publication January 1, 1964

IN recent years various attempts have been made at estimating incidence rates for malignant neoplasms among African populations, as in Johannesburg by Higginson and Oettlé (1960), and in Uganda, mainly around Kampala, by Davies (1961) and Knowelden (1957, 1963). Such studies have mainly been carried out in urban areas while an estimate of cancer incidence in rural areas has only been attempted in a collaborative effort between The University of Louvain and The Danish Cancer Registry supported by the Anna Fuller Fund and the National Institutes of Health, U.S.A., as preliminarily reported by Gigase, Clemmesen and Maisin (1962).

A special lymphoma has been studied by Burkitt (1958, 1962) and associates in a team work carried out from Kampala, and Kaposi's disease by Lothe (1963).

The high incidence among the Chinese people of nasopharyngeal carcinoma, which has been known for several years, was first reported by Maxwell (1929) and later confirmed by Dunlap (1938). Digby, Fook and Che (1941) reported that in Hong Kong nasopharyngeal carcinomas were the second commonest variety of malignant diseases.

In Cuba the high incidence of rhinopharynx cancer was confirmed among Chinese emmigrants by Martinez (1940).

In Java, Bonne (1937) reported a great number of these tumours among Javanese in Batavia which corresponds with reports of Kouwenaar (1950) and Sutomo (1950). The incidence of nasopharyngeal tumours in Malaya has been demonstrated by Marsden (1958) who showed that not only Chinese but also Malays have a higher proportion of these tumours, and this has been confirmed by Djojopranoto and Marchetta (1959).

In the Philippines, Hasselmann (1934) and Bonne (1937) have reported on the high incidence of nasopharyngeal tumours.

In contrast to the high incidence of this malignancy among the races mentioned above Godtfredsen (1947) found a low ratio of nasopharyngeal neoplasms in Denmark and Sweden in relation to all cancer. This corresponds with the findings of Molony (1957) in Canada who gave an estimate of 0·3 per cent of all malignant diseases. The same low ratio has been found by Das *et al.* (1954) for India, by Daito, Sakamoto and Hara (1952) for Japan, and by Martin and Quan (1951), who doubted whether any prediliction for post nasal space tumours extended to oriental races.

A higher occurrence of post nasal space tumours among hospital patients in

* Present address.

Nairobi was reported by Clifford (1961a) which has directed attention to the frequency and possible aetiology of this neoplasm among the African population.

MATERIAL

The original material surveyed comprises 136 clinical cases from the Department of Head and Neck Surgery, King George VI Hospital, Nairobi. All cases have been confirmed as malignant by biopsy. Of this material 97 were found to have been diagnosed as tumours of the post nasal space as defined by Clifford (1961a). The remaining 39 tumours consist of 36 malignant neoplasms of the upper respiratory tract without precisely located origin in this region. Three appeared to be tumours of other origin.

FIG. 1.—Shows the total number of cases during 1957–62 divided into year and sex.

Ninety-seven cases of malignant neoplasms of the post nasal space covering a 6-year period 1957–62 were gathered from ward sheets from King George VI Hospital and from the cancer registry of the Medical Research Laboratory.

Fig. 1 shows the annual number of cases by sex. It is seen that the number of patients is increasing during the period studied, more so for males than for females.

Table I shows the age distribution of 55 cases of carcinoma of the post nasal space. The maximum for both sexes is in the age group of 25–34 years ; it must be emphasised, however, that the quality of the material does not allow a more detailed grouping as many of the patients do not know their age exactly.

A histological review of the post nasal space tumours concerned showed full agreement with regard to nomenclature and evaluation of findings.

Table II gives the histological distribution of the 97 cases. It appears that nearly 50 per cent consists of dedifferentiated epidermoid carcinomas. Of 75 cases a well defined number of eosinophil granulocytes were present in 16 cases.

TABLE I.—*Tentative Age Distribution of 55 Carcinomas of the Post Nasal Space Tumours seen in King George VI Hospital 1957–62*

| Age groups | Number of cases |
|---|---|
| 0–14 | 0 |
| 15–24 | 11 |
| 25–34 | 13 |
| 35–44 | 12 |
| 45–54 | 11 |
| 55–64 | 5 |
| 65–74 | |
| 75–85 | 1 |
| Total | 55 |

All biopsies were taken as described by Clifford *et al.* (1963) by a Luc's forceps introduced through the nose.

TABLE II.—*Distribution of 97 Post Nasal Space Tumours in Kenya 1957–62 According to Histological Type*

| | |
|---|---|
| Carcinomas : | |
| Dedifferentiated epidermoid carcinomas | 52 |
| Differentiated epidermoid carcinomas | 9 |
| Adenocarcinomas | 5 |
| Anaplastic carcinomas | 16 |
| Others | 1 |
| Sarcomas : | |
| Reticulosarcomas | 5 |
| Lymphosarcomas | 3 |
| Malignancy unspecified | 6 |
| Total | 97 |

It was only possible to register the duration of illness for the patients who died in hospital a known time from the beginning of the disease. This was recorded for 25 patients, 18 males and 7 females. The average duration of illness was found to be 15 months for males and 12 months for females.

The symptoms most common among the patients are given in Table III.

TABLE III.—*The most Common Symptoms among 97 Patients with Tumours of the Post Nasal Space*

| Symptoms | Number of cases |
|---|---|
| Swelling of the right side of neck | 35 |
| of these, small lumps left side | 13 |
| Headache | 38 |
| Swelling of the left side of neck | 23 |
| of these, small lumps right side | 7 |
| Proptosis of the eye and involvement of cranial nerves | 32 |
| Symptoms from the ear as earache, slight deafness, impaired hearing or pain | 29 |
| Epistaxis | 25 |
| Discharge from the nose and/or difficulties in breathing through the nose | 28 |
| Dysphagia | 19 |
| Trismus | 13 |
| Cough | 7 |
| Tumour infiltration of the soft palate | 8 |
| Blood in sputum | 8 |
| Hoarseness of voice | 1 |

Among the patients with severe headache about one-third had radiographically demonstrated destruction of the base of skull. It is noticed from Table III that tumour infiltration of the orbit and metastasis to regional lymph glands in the neck are the most common symptoms. It also appears that most patients arrive at the hospital with severe advanced symptoms. The duration of illness before arrival at King George VI Hospital was known for 75 of the 97 patients. The average time was 9 months ranging between 2 weeks and $2\frac{1}{2}$ years.

Radiological examination showed evidence of abnormalities in the upper respiratory tract in 41 cases. Of these 19 had radiologically demonstrated erosion of base of skull and 11 had soft tissue swellings of the retropharyngeal space.

As X-ray therapy is not available in East Africa 68 patients were treated with cytotoxic agents. The drugs used were mainly nitrogen mustard, chloramine mustard, dimethylmyleran, and 5-fluorodeoxyuridine. Valuable results of this therapy have recently been reported by Clifford *et al.* (1963).

Autopsy reports have been studied in 20 cases. The main findings in these are given below :

| | |
|---|---|
| Erosion of base of skull by tumour infiltration . | 9 |
| Tumour in the post nasal space . . . . | 15 |
| Metastasis in lungs/mediastinum . . . . | 3 |
| ,, ,, liver . . . . . . | 2 |

Among the cases with erosion of the base of skull one showed meningitis carcinomatosa.

*Incidence*

The frequency of post nasal space tumours and the total number of neoplasms in Kenya during the 6-year period 1957–1962 inclusive are shown in Table IV. From the figures it is evident that the number of post nasal space tumours is increasing much more than the total number of malignant diseases, which might be accounted for by the special interest and attention shown to this neoplasm.

TABLE IV.—*The Annual Occurrence of Post Nasal Space Tumours in Kenya Compared with the Annual Number of Malignant Diseases Registered* 1957–1962

| | Post nasal space tumours | | | All cancers | | |
|---|---|---|---|---|---|---|
| Year | Males | Females | Total | Males | Females | Total |
| 1957 . . . | 7 | 0 | 7 | 259 | 209 | 468 |
| 1958 . . . | 4 | 1 | 5 | 365 | 208 | 573 |
| 1959 . . . | 10 | 2 | 12 | 277 | 246 | 523 |
| 1960 . . . | 11 | 4 | 15 | 307 | 271 | 578 |
| 1961 . . . | 16 | 11 | 27 | 347 | 274 | 621 |
| 1962 . . . | 26 | 5 | 31 | 389 | 333 | 722 |
| Total . . | 74 | 23 | 97 | 1944 | 1541 | 3485 |

The tribal distribution of rhinopharynx tumours is shown in Table V. The incidence calculations are based on population figures from the census in 1948. It appears from the table that the largest number of cases is found among the Kikuyus, which is the most numerous and advanced tribe. In addition, this tribe is favoured by good medical facilities, as the main part lives near Nairobi. The only tribe which shows figures different from those of other tribes is the Nandis. The main area where this tribe lives is not far from Kisumu where also good medical facilities are available. An incidence of 1·43 per 100,000 is however remarkable.

TABLE V.—*Distribution of* 97 *Post Nasal Space Tumours by Tribe seen in King George VI Hospital, Nairobi,* 1957–1962

| Tribe | Males | Females | Total | Average annual incidence per 100,000 |
|---|---|---|---|---|
| Kikuyu | 24 | 7 | 31 | 0·50 |
| Nandi | 8 | 2 | 10 | 1·43 |
| Kamba | 7 | 2 | 9 | 0·25 |
| Luo | 6 | 2 | 8 | 0·18 |
| Meru | 3 | 4 | 7 | 0·36 |
| Baluhya | 6 | 0 | 6 | 0·15 |
| Kisii | 4 | 1 | 5 | 0·33 |
| Kipsigis | 2 | 3 | 5 | 0·52 |
| Embu | 2 | 0 | 2 | 0·16 |
| Others | 12 | 2 | 14 | 0·28 |
| Total | 74 | 23 | 97 | 0·31 |

Table VI gives the incidence of neoplasms of the upper respiratory tract other than the post nasal space. From this table calculated on the same population figures as Table V (census 1948) the Nandi group appears to have a rate of 0·57 which is not different from the other tribes.

TABLE VI.—*The Annual Incidence and Distribution by Tribe of Cases of Malignant Neoplasms of the Antrum/Maxilla, Tongue, Oral Cavity, Larynx, Tonsils, Palate, Pharynx,* (*Unspecified*) 76 *Cases in All, and* 36 *Cases of the Upper Respiratory Tract,* (*Unspecified*) *Other than Post Nasal Space Tumours, seen in King George VI Hospital* 1957–62

| | Antrum/Maxilla and as mentioned above | | | Site unspecified | | | | |
|---|---|---|---|---|---|---|---|---|
| Tribe | Males | Females | Total | Males | Females | Total | Total | Average annual incidence per 100,000 1957–62 |
| Kikuyu | 3 | 18 | 21 | 6 | 5 | 11 | 32 | 0·52 |
| Luo | 6 | 2 | 8 | 2 | 1 | 3 | 11 | 0·24 |
| Baluhya | 1 | 2 | 3 | 0 | 1 | 1 | 4 | 0·10 |
| Kamba | 3 | 2 | 5 | 4 | 0 | 4 | 9 | 0·25 |
| Meru | 1 | 0 | 1 | 1 | 1 | 2 | 3 | 0·15 |
| Kisii | 1 | 2 | 3 | 1 | 2 | 3 | 6 | 0·39 |
| Embu | 0 | 0 | 0 | 1 | 0 | 1 | 1 | 0·08 |
| Kipsigis | 1 | 0 | 1 | 3 | 0 | 3 | 4 | 0·42 |
| Nandi | 2 | 0 | 2 | 2 | 0 | 2 | 4 | 0·57 |
| Others/Unknown | 20 | 12 | 32 | 3 | 3 | 6 | 38 | 0·75 |
| Total | 38 | 38 | 76 | 23 | 13 | 36 | 112 | 0·36 |

The frequency of the post nasal space tumours in Kenya, Uganda,* and Johannesburg is shown in Table VII. The figures used for calculating the inci-

* The Uganda material originated from data from the Kampala Cancer Registry and ward sheets from the Mulago Hospital, Kampala. All cases have had malignancy confirmed by biopsy, though some only from neck metastases.

dence in Uganda were taken from the census of 1959. The numbers of cases used in the calculation originate from the Mengo district, during the period 1957–1962 inclusive.

TABLE VII.—*Incidence of Post Nasal Space Tumours in Kenya, Uganda and Johannesburg Compared with Ratio to all Cancer in the Same Districts*

| Districts | Number of cases with post nasal space tumours | Population | Average annual incidence of post nasal space tumours per 100,000 | All cancers registered | Average annual incidence of all cancers per 100,000 |
|---|---|---|---|---|---|
| Kenya | 73 (1960–62) | 8,200,000* | 0·30 | 1960–62 : 1·921 cases | 7·81 |
| Uganda : | | | | | |
| Mengo district | 26 (1957–62) | 1,294,879 | 0·33 | † 1957–62 : 1·697 cases | 43·68 |
| All Uganda | 41 (1957–62) | 6,449,558 | 0·11 | | 8·77 |
| Johannesburg | 8 (1953–55) | 478,464 | 0·56 | 1953–55 : 955 cases | 66·53 |

* The African population of 8,200,000 is calculated from a preliminary report of the census of 1962.

† Most cancers counted in the Kampala registry originate from the Mengo district, being patients who have consulted Mulago Hospital, Kampala. Some cases, however, do come from outside this area.

From Table VII it appears that the incidence rate of total cancer for Johannesburg, 66·53, is the highest. The rate for the Mengo district, Uganda, is of the same order of magnitude 43·68. The far lower incidence for Uganda as a whole, 8·77, corresponds in magnitude to the rate for Kenya as a whole, 7·81, and indicates that the efficiency of diagnosis for the country leaves something to be desired in comparison to urban areas as Johannesburg and the Mengo district around Kampala.

These general conditions should be kept in mind in evaluating the incidence rates for the post nasal space tumours, which in Kenya are of the same order of magnitude as in Uganda, while Johannesburg shows slightly higher values.

From Table VIII showing the histological pattern of the post nasal space tumours in Kenya, Uganda and Johannesburg ; it appears that in all three areas carcinomas are the most frequent.

TABLE VIII.—*The Histological Pattern of the Post Nasal Space Tumours in Kenya, Uganda and Johannesburg*

| Country | Carcinomas | Sarcomas | Unclassified malignant tumours | Total |
|---|---|---|---|---|
| Kenya | 83 | 8 | 6 | 97 |
| Uganda | 30 | 7 | 4 | 41 |
| Johannesburg | 8 | 0 | 0 | 8 |

Table IX shows the occurrence of neoplasms of the antrum/maxilla, post nasal space, palate, tonsils, tongue, and oral cavity in Kenya and Uganda in the African population during the years 1957–1962 inclusive. It suggests a greater number of post nasal space tumours in Kenya than in Uganda in accordance with the explanation given. The difference for tumours of the palate is easily explained by the difference in diagnostic methods.

FIG. 2.—Shows the geographical distribution of 33 cases of post nasal space tumours.

TABLE IX.—*The Annual Occurrence of Neoplasms of the Maxilla/Antrum, Post Nasal Space, Palate, Tonsils, Tongue and Oral Cavity in Kenya and Uganda among the African Population during the 6-year Period* 1957–1962

| | Maxilla/ Antrum | Post nasal space | Palate | Tonsils | Tongue | Oral cavity | Total |
|---|---|---|---|---|---|---|---|
| Kenya | 18 | 97 | 5 | 5 | 22 | 13 | 160 |
| Uganda | 22 | 41 | 23 | 14 | 14 | 12 | 126 |

*Geographical epidemiology*

The geographical epidemiological studies of the post nasal space tumours were carried out for 33 patients. The addresses have been plotted on a map, which at the same time gives the population density. From Fig. 2 it appears that the cases are generally found in the areas of the greatest population density.

*Aetiology*

As a possible factor of aetiological significance to the post nasal space neoplasms in Kenya, fumes and smoke from cooking fires inside the African huts has been suggested by Clifford (1961b, c). With regard to the tumours among Chinese patients this aetiology was also mentioned by Digby *et al.* (1941). Certain fumes are known to be carcinogenic, and the air pollution inside huts with bad ventilation and without chimneys might be considerable. Some huts were visited by the author to obtain an impression of the air pollution. Sometimes an open fire was placed on the floor inside the hut itself; but the fireplace might be situated in different ways, such as in the middle of the room or along the wall in close connection with a window. With regard to ventilation a common finding was a big gap ranging 20–50 centimetres in size between the roof and the wall. This together with considerable leakiness offered good possibilities of effective ventilation. In all huts visited soot was found hanging down from the roof mixed with a considerable amount of spider webs and dirt. In only one hut was heavy smoke and air pollution found on arrival. The average age of an African hut is about 5 years, and soot seems to be allowed to deposit for the lifetime of the hut. The fireplace is mainly used for cooking, in which occupation only women are employed. From Fig. 1 it is seen that most of the patients are males.

The 12 patients alive were interviewed with regard to how many hours in 24 the fireplace was working. The average finding was 5 hours.

The incidence of lung cancer was registered during the same period as the tumours of the post nasal space. From Table X it appears that the annual number of lung cancers is rather constant and the frequency is not increasing or in any way parallel to the tumours of the post nasal space.

TABLE X.—*Lung Cancer in Kenya Registered During the Years* 1957–1962

| | 1957 | 1958 | 1959 | 1960 | 1961 | 1962 | Total |
|---|---|---|---|---|---|---|---|
| Males | 3 | 4 | 1 | 7 | 3 | 5 | 23 |
| Females | 1 | 1 | 2 | 1 | 1 | 1 | 7 |
| Total | 4 | 5 | 3 | 8 | 4 | 6 | 30 |

With regard to smoke inside the huts a comparative investigation between the island of Zanzibar and the Kenya population might be of interest as the huts on Zanzibar have a special shelter for cooking which is not used for sleeping or other purposes than cooking.

Capps (1950) and Winborn (1955) have mentioned allergic and suppurative rhinitis as a possible irritative factor producing metaplasia of the epithelium of the rhinopharynx and the sinuses. As previously mentioned, a considerable number of eosinophil granulocytes were present in 16 cases. Bearing in mind that most of the patients consult the hospital very late in the disease, no conclusions can be drawn from the material with regard to this possible aetiology.

Referring to the high incidence of neoplasms of the post nasal space among Chinese in China as well as the Chinese immigrants in U.S.A. who are exposed to different environmental factors, an inherited racial predisposition of a genetic nature may be suggested as significant in considering the aetiology of this tumour. With regard to the increased rate among the Malays this point has been emphasised by Marsden (1958).

In the view of the author snuff may be one of the most important aetiological factors in tumours of the post nasal space in Kenya. As pointed out by Shapiro *et al.* (1955), Davies (1961) and Keen (1963), the inhalation of snuff is a common African habit in consuming tobacco. Snuff is used by males as well as by females. Shapiro *et al.* (1955) has suggested that the comparative incidence of antral neoplasm in the Bantu and lung cancer in the European can be accounted for by their different tobacco habits.

From Cooper and Campbell's (1955) chemical analysis of Zulu and Venda snuff it appears that 3,4-benzopyrene is present in a considerable amount together with other carcinogenic hydrocarbons.

Twelve patients with tumours of the post nasal space were interviewed with regard to their habits of tobacco consumption. From Table XI it is seen that six of the patients used snuff and three of them cigarettes. With regard to the reliability of the negative answers it is worth mentioning that it is not considered appropriate to advanced Africans to use snuff, and that its use varies considerably throughout Kenya.

TABLE XI.—*Tobacco Habits of* 12 *Patients with Carcinoma of the Post Nasal Space*

| Kind of tobacco used | Number of patients |
|---|---|
| Snuff | 6 |
| Cigarettes | 3 |
| Chewing tobacco | 1 |
| None | 2 |
| Total | 12 |

Most tribes in East Africa use snuff as a general way of tobacco consumption ; liquid snuff is common only among certain tribes. From Huntingford's studies (1950) it is seen that among Nandies as well as among the Kipsigis, living nearly in the same area, it is quite a common custom to use liquid snuff. This might be supposed to contain a soluble carcinogen responsible for the increased incidence of post nasal space tumours among these tribes. From Table V it appears that the annual incidence rate for the Nandis is 1·43 and for the Kipsigis 0·52, which is remarkably high. Bearing in mind the use of liquid snuff this factor might be worth investigating in the future with regard to the aetiology of the Kenya cases.

SUMMARY

Ninety-seven cases of post nasal space tumours from King George VI Hospital, Nairobi, among Africans in Kenya are reviewed with regard to duration of illness, symptoms, X-ray findings and pathology. As measured by cases known from the cancer registries and hospitals the incidence of these tumours is the same in Kenya, Uganda and Johannesburg. Only the Nandi tribe is found to have a high annual incidence, 1·43, in comparison with other tribes. The geographical distribution is shown. The aetiology is discussed, and snuff is suggested as a possible agent which is considered worth investigating in the future.

In consequence of the limited number of cases, as well as of the difficulties in collecting material under African conditions, the results presented should be treated with the same caution as other studies from these regions.

This investigation was supported by The Anna Fuller Fund, U.S.A.

The author wishes to thank Mr. Peter Clifford, head of the department of Head and Neck Surgery, King George VI Hospital, Nairobi, and Dr. A. Linsell, head of the Medical Research Laboratory, Nairobi, for kind interest and working facilities. Due to very efficient co-operation with Professor Hutt, Department of Pathology, Makerere College, Kampala, and Mrs. Barbara Stroud, of the Kampala Cancer Registry, it was possible to carry out studies there. The author also wishes to thank Dr. A. G. Oettle for his excellent help. He kindly put at my disposal his material from his study from 1953–55 in Johannesburg.

Finally, thanks are due to Dr. Johannes Clemmesen, The Danish Cancer Registry and the Department of Pathology of the Finsen Institute, Copenhagen, for interest and help in theory as well as in the field.

### REFERENCES

Bonne, C.—(1937) *Amer. J. Cancer*, **30**, 435.

Burkitt, D.—(1958) *Brit. J. Surg.*, **46**, 218.—(1962) *Post Grad. med. J.*, **38**, 71.

Capps, F. C. W.—(1950) *Proc. Roy. Soc. Med.*, **43**, 665.

Clifford, P.—(1961a) *E. Afr. med. J.*, **38**, 491.—(1961b) *Brit. J. Surg.*, **48**, 15.—(1961c) *J. Laryng.*, **75**, 707.

*Idem*, Oettgen, H. G., Beecher, J. L., Brown, F. P., Harries, J. R. and Lawes, W. E.—(1963) *Brit. med. J.*, i, 1256.

Cooper, R. L. and Campbell, J. M.—(1955) *Brit. J. Cancer*, **9**, 528.

Daito, T., Sakamoto, H. and Hara, H. J.—(1952) *Arch. Otolaryng., Chicago*, **56**, 46.

Das, T., Taneja, G. M., Chaddah, M. R. and Minocha, D. B.—(1954) *Ann. Otol., etc., St. Louis*, **63**, 890.

Davies, J. N. P.—(1961) *E. Afr. med. J.*, **38**, 486.

Digby, K. H., Fook, W. L. and Che, Y. T.—(1941) *Brit. J. Surg.*, **28**, 517.

Djojopranoto, M. and Marchetta, F. C.—(1959) *Arch. Otolaryng., Chicago*, **69**, 155.

Dunlap, A. M.—(1938) *Chin. med. J.*, **53**, 68.

Gigase, P., Clemmesen, J. and Maisin, J.—(1962) ' Cancer in Kivu and Rwanda-Urundi ', Louvain (Université de Louvain, Institut du Cancer).

Godtfredsen, E.—(1947) *Brit. J. Ophthal.*, **31**, 78.

Hasselmann, C. M.—(1934) *Philipp. J. Sci.*, **54**, 1.

Higginson, J. and Oettlé, A. G.—(1960) *J. nat. Cancer Inst.*, **24**, 589.

Huntingford, G. W. B.—(1950) ' Nandi Work and Culture ', London (Her Majesty's Stationery Office for the Colonies).

KEEN, P.—(1963) *Clin. Radiol.*, **14**, 250.
KNOWELDEN, J.—(1957) *Proc. Roy. Soc. Med.*, **50**, 249.—(1963) *Ibid.*, **56**, 529.
KOUWENAAR, W.—(1950) ' On Cancer Incidence in Indonesia '. Symposium on Geographical Pathology and Demography of Cancer, Oxford, England.
LOTHE, F.—(1963) *Acta path. microbiol. scand.*, Suppl. 161.
MARSDEN, A. T. H.—(1958) *Brit. J. Cancer*, **12**, 161.
MARTIN, H. AND QUAN, S.—(1951) *Ann. Otol., etc., St. Louis*, **60**, 168.
MARTINEZ, E.—(1940) *Bol. Liga Cáncer, Habana*, **15**, 276.
MAXWELL, J. L.—(1929) *Chin. med. J.*, **43**, 462.
MOLONY, T. J.—(1957) *Laryngoscope*, **67**, 1297.
SHAPIRO, M. P., KEEN, P., COHEN, L. AND DE MOOR, N. G.—(1955) *S. Afr. med. J.*, **29**, 95.
SUTOMO, T.—(1950) ' Additional data on cancer Incidence in Indonesia '. Symposium on Geographical Pathology and Demography of Cancer, Oxford, England.
WINBORN, C. D.—(1955) *Arch. Otolaryng., Chicago*, **61**, 141.